# The challenge of assessing invasive biomarkers for epilepsy surgery

**DOI:** 10.1093/brain/awae164

**Published:** 2024-05-16

**Authors:** Nicolas Roehri, Serge Vulliemoz, Stanislas Lagarde

**Affiliations:** EEG and Epilepsy Unit, University Hospitals and Faculty of Medicine, University of Geneva, CH-1211 Geneva, Switzerland; EEG and Epilepsy Unit, University Hospitals and Faculty of Medicine, University of Geneva, CH-1211 Geneva, Switzerland; Center for Biomedical Imaging (CIBM), Lausanne and Geneva, 1015 Lausanne, Switzerland; EEG and Epilepsy Unit, University Hospitals and Faculty of Medicine, University of Geneva, CH-1211 Geneva, Switzerland; APHM, Timone Hospital, Epileptology and Cerebral Rhythmology, 13005 Marseille, France; Aix Marseille Univ, INSERM, INS, Inst Neurosci Syst, 13005 Marseille, France

We read with interest the article that has recently been published by *Brain* entitled ‘Spike ripples localize the epileptogenic zone best: an international intracranial study’ by Shi *et al.*^1^ This study examined a substantial dataset comprising over 100 patients from various centres, using an automated algorithm designed to detect interictal spike and ripple events, collectively referred to as ‘spike ripple,’ in intracranial EEG recordings. The aim of this study is to investigate whether this biomarker outperforms other existing biomarkers in accurately localizing the epileptogenic zone. Notably, the authors have integrated data from both spikes and ripples, aligning with our prior study's recommendation, which underscored the complementary nature of spikes and high-frequency oscillations (HFOs).^2^ Such studies are crucial in the search for the most effective interictal biomarker for epileptogenicity.

We have, however, some concerns about how this manuscript presents non-statistically significant results, as it could potentially lead to misinterpretation of the study’s findings. Additionally, the rate ratio metric and certain statistical analyses used appear inappropriate, which could introduce bias into certain results. We would like to raise the following specific methodological issues.

First, in the abstract and corresponding section of the results, the authors present some findings as evidence despite their lack of statistical significance.

  ‘Subjects with curative resection (ILAE 1) had a higher proportion of spike ripple generating brain tissue removed compared to those who were not seizure free (ILAE 2–6; *P* = 0.06, d = 0.17).’

‘The percentage of ILAE 1 subjects with the majority of spike ripples removed was higher than the percentage of subjects with the majority of spikes (69%, *P* = 0.12), spike-gamma (69%, *P* = 0.12), wideband HFOs (63%, *P* = 0.03), ripples (45%, *P* = 0.01), or fast ripples (36%, *P* < 0.001) removed.’

These excerpts illustrate the authors’ claims. They first claim that a higher proportion of spike ripples were removed in patients with a favourable outcome compared to those with a poor outcome, despite not being statistically significant (*P* = 0.06) and a low effect-size (*d* = 0.17, equivalent to an area under the curve of 0.585). Their second claim is that there was a higher proportion of good outcome patients with the majority of spike ripples removed compared to spikes or spike-gamma, again without statistically significant evidence (*P* = 0.12). We acknowledge that *P*-values and null-hypothesis significance testing have limitations due to their overly dichotomous nature. However, stating that there is an effect with such *P*-values and low effect size is clearly misleading. A more accurate conclusion would have acknowledged the absence of statistically significant differences between patients with favourable and unfavourable outcomes regarding the proportion of spike ripple-generating brain tissue removed. Additionally, it should have explicitly stated that the proportion of ILAE 1 subjects with the majority of spike ripples removed was not statistically higher than for spikes and spike-gamma. Furthermore, none of the aforementioned tests related to the percentage of ILAE 1 subjects with the majority of spike ripples removed have been corrected for multiple comparisons, a step that should have been taken given that the results of the spike ripple biomarker have been compared to all the other biomarkers. For instance, applying Bonferroni or false discovery rate correction would render the comparison between spike ripple and wideband HFOs not significant.

Second, the event rate ratio (RR) used here to quantify the different biomarkers, while common in the literature, is debatable. In fact, it solely evaluates specificity to the resected volume, lacks clinical applicability and sometimes produces counter-intuitive results, which contradict those obtained through other commonly used metrics from the binary classification field. Figure 1 illustrates these points with six toy examples. It also shows the classification performance with and without considering the imbalance between resected and non-resected channels.

**Figure 1:**
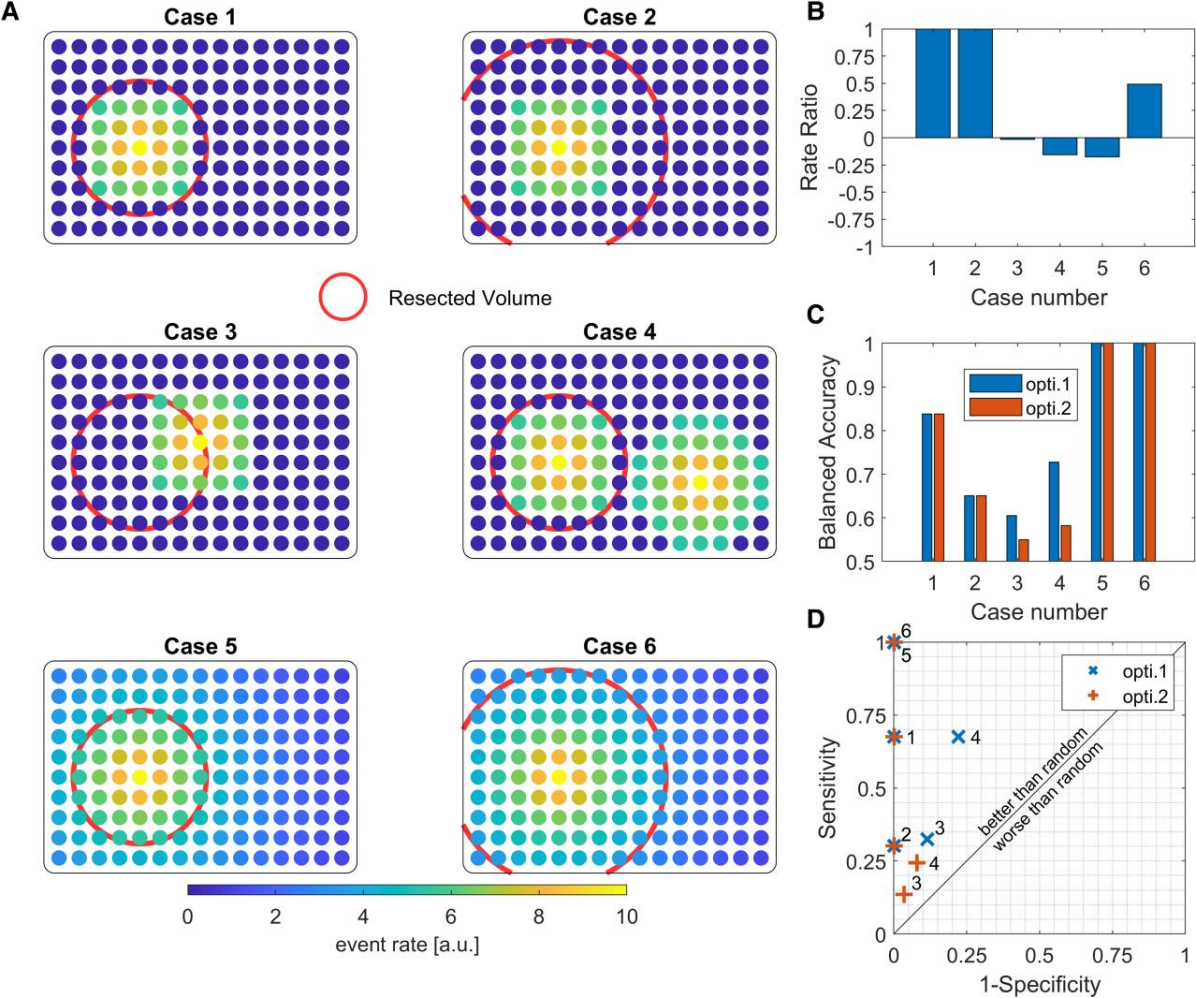
**Visualization of six toy examples used to compare the event rate ratio and balanced accuracy.** For each case, dots represent intracranial channels (visually arranged on a 2D sheet for clarity), colour-coded by their event rates. The resected volume (RV) is outlined by a red circle (**A**). Bar plots display the event rate ratio (RR) and balanced accuracy (bACC) for each case (**B** and **C**, respectively). Sensitivity and specificity for each case are depicted in the receiver operating characteristic (ROC) space (**D**). Sensitivity represents the percentage of correctly predicted resected channels, while specificity represents the percentage of correctly predicted non-resected channels. The average of sensitivity and specificity, bACC, is calculated considering two optimization approaches (opti.) (**C**) Optimization 1 does not account for the proportion of resected over non-resected channels, while Optimization 2 does.^3^ Cases 1 and 2 exhibit high event rates within the resected volume and zero outside, with Case 2 having a larger RV than Case 1. Despite both Cases having a rate ratio and specificity values of 1, the bACC and sensitivity is lower in Case 2. Cases 3 and 4 show scenarios where the RR is negative, because either the events are present at the border of the RV or there are two event generating zones, one inside and a stronger one outside the RV. The event distribution would suggest the need for resection enlargement in patients with poor outcomes. In such situations, bACC, sensitivity and specificity vary depending on optimization approaches. Optimization 1 clearly overestimates the performance of the metric, emphasizing the importance of considering the proportion of resected over non-resected channels. In Case 5, the RR is negative but the bACC, sensitivity and specificity values equal 1. This means that the RR is negatively biased by the extension of the event even though the RV contains the channels with highest rates. Case 6 is similar to Case 5, except that the RV is larger, making the RR positive.

An ideal biomarker is not only specific but also sensitive.^4,5^ The hope of finding a biomarker in the current context is either to better delineate the epileptogenic zone (EZ) to improve surgical outcome or to predict the surgical outcome. A biomarker with perfect specificity but poor sensitivity would only delineate a portion of the EZ, and removing partially the EZ would not lead to seizure freedom. For instance, if spike ripples are found only in one-third of the electrodes implanted in the EZ and nowhere else, its sensitivity would be 30% but its RR would equal 1 (Fig. 1A, Case 1 versus Case 2). This may account for the lack of statistical difference in the RR of the spike ripples between patients with favourable and unfavourable outcomes. It is plausible that the most epileptogenic areas, i.e. with the highest event rates, were removed in the latter group, in line with the intention-to-treat principle. Alternatively, the authors’ hypothesis posits that a high RR in patients with poor outcome could arise from incomplete coverage of the EZ (Fig. 1D in Shi *et al*.^1^). If this were true, any invasive biomarker would be inefficient in such scenarios, as acknowledged by the authors. Moreover, clinicians had sufficient other information from both non-invasive and invasive investigations to propose surgery, thereby lowering the likelihood of this hypothesis in most of the patients.

Even if a biomarker were able to differentiate between the two patient groups based on its RR, it remains unclear how the RR would influence the clinical decision making. A biomarker with an RR close to zero or negative could indicate the need to enlarge the resection, only if the event rate is high outside the resected volume (Fig. 1A, Case 3 and 4). However, it may not aid in delineation or ensure seizure freedom, as its sensitivity has not been evaluated. In other words, removing the remaining area with high event rates may not suffice, as this biomarker may not be present in all regions of the EZ. This risk of high specificity but low sensitivity was already acknowledged for fast ripples,^2,6,7^ which greatly limited their clinical use. It is possible that removing the majority of spike ripples is necessary but not sufficient to guarantee seizure freedom, again mirroring earlier research on fast ripples.^8^ In some cases, the RR could also be negative, even if all channels with the highest rates are within the resected volume (i.e. perfect separation resected versus non-resected channels, balanced accuracy equals 1). This may occur due to the widespread distribution of events (e.g. epileptic spikes) with high rates within the resected volume but lower rates outside the resected volume (Fig. 1A, Case 5). The RR is thus negatively biased by the extent of the event, could underestimate its performance, and could contradict other binary classification metrics.

Finally, the authors seem to have employed statistical tests designed for independent groups on dependent groups. The methods section clearly states that spike ripples, spikes, spike-gamma and HFO were ‘detected on all data from all sites’ (sites A, B, C and D).

Right-tailed two-proportion *z*-test is used to determine if the proportions of categories in two group variables significantly differ from each other. However, this test requires that the two groups are independent, yet in this article, the proportions being tested come from the same group of patients but are characterized by different biomarkers, thus violating the assumption of independence. We believe that the McNemar’s test would have been more suitable, as it is designed to compare proportions between two dependent populations with paired samples. Similarly, while Cliff’s *d*-value quantifies effect size related to the Mann–Whitney U-test or Wilcoxon rank-sum test for independent populations, it is inadequate for comparing different biomarkers in patients with favourable outcomes. Cohen’s *d* effect size calculated for paired sample (if the assumption of normality is met) would have been more adequate. With appropriate tests, the previously non-significant results could have potentially reached significance, as they would have been able to quantify more subtle increases in performance.

In conclusion, due to the previously discussed limitations regarding the statistical methodology, it appears challenging to fully support the authors’ claims regarding the differences in spike ripples between patients with favourable and unfavourable outcomes, and their assertion that spike ripples are a superior biomarker compared to spikes, spike gamma and HFOs. The study certainly adds valuable information to this important field of research but presenting results as evidence despite their lack of statistical significance and low effect size is misleading, particularly as these findings may linger and influence future studies. This could be detrimental to the research domain and, ultimately, the clinical practice. Even more crucially, the examples depicted in Fig. 1 underscore the challenge of identifying an optimal metric for assessing the performance of biomarkers for epilepsy surgery. It is essential to emphasize the importance of utilising metrics that account for both sensitivity and specificity/precision, as well as the inherent imbalance between positive (i.e. small EZ) and negative classes (i.e. larger non-EZ), which could be patient dependent. Further research and discussion on quantifying the performance of biomarkers will be pivotal for advancing this research field and enhancing its clinical applicability.

## Data Availability

The code used to produce Fig. 1 is available at https://gitlab.unige.ch/vulliemoz_group/toy_examples/-/blob/main/Event_Rate_Ratio_vs_Accuracy.
